# Correlation of preoperative frailty with postoperative delirium and 1-year mortality in Chinese geriatric patients undergoing non-cardiac surgery: a prospective observational cohort study

**DOI:** 10.1097/JS9.0000000000002042

**Published:** 2024-08-14

**Authors:** Min Zhang, Xiaojun Gao, Mengjie Liu, Zhongquan Gao, Yongle Guo, Lina Chen, Yang Liu, Xiaoning Zhang, Linlin Huang, Meng Tong, Ting Zou, Yongtao Sun

**Affiliations:** a Department of Anesthesiology, The First Affiliated Hospital of Shandong First Medical University & Shandong Provincial Qianfoshan Hospital; b Shandong Institute of Anesthesia and Respiratory Critical Medicine; c Shandong Provincial Clinical Research Center for Anesthesiology; d Department of Anesthesiology, Jinan Materrity and Child Care Affiliated to Shandong First Medical University; e Shandong First Medical University & Shandong Academy of Medical Sciences; f Department of Nursing, The First Affiliated Hospital of Shandong First Medical University & Shandong Provincial Qianfoshan Hospital, Jinan, China

Highlights

- This is the first study that will examine the association between preoperative assessment of frailty and the occurrence of POD after non-cardiac surgery.
- This single-center prospective observational cohort study provides new insights into the prevention of POD after non-cardiac surgery in frail patients.
- In view of the low educational level of the elderly over 70 years old in China, we will use MMES to perform stratified evaluation according to their educational level before surgery.
- The mFI has been extensively studied in surgical patients 70 years of age or older, although it has rarely been used to assess frailty among patients undergoing non-cardiac surgery.
- Preoperative frailty was an independent risk factor for postoperative delirium and increased the incidence of adverse events 1 year after surgery, but it did not increase the 1-year all-cause mortality or the readmission rate.

Geriatric adults often suffer from delirium, an acute disorder of attention and cognition that can be life-threatening^1^. Geriatric patients are undergoing surgery more frequently over the last 20 years, and elderly people get sick more often than others^2^. Frailty is more prevalent in old adults after surgery (frailty percentage 42–50%) than in the nonsurgical aged population (frailty proportion 4–10%)^3^.

Frailty in elderly individuals is also associated with lower quality of life and is an accurate predictor of all-cause mortality^4^. The therapeutic value of preoperative decision-making and prognostic assessment depends on the early detection of frailty in patients. Preoperative frailty has not yet been proven to be a separate risk factor for POD^5^. Therefore, this study investigated the relationship between preoperative frailty and the incidence of POD and 1-year mortality in elderly Chinese patients after non-cardiac surgery.

This single-center, prospective, observational cohort trial ran between 7 February 2022 and 31 December 2023 at the First Affiliated Hospital of Shandong First Medical University, China. This trial was approved by the Ethics Committee of the First Affiliated Hospital of Shandong First Medical University on 23 November 2021. All patients provided written informed consent before participation in the study procedures. The work has been reported in line with the STROCSS criteria.

Patients were included if they were scheduled to receive elective non-cardiac surgery, were aged older than or equal to 70 years, had an American Society of Anesthesiologists (ASA) grade of I–IV, and had an expected hospital stay longer than 3 days after surgery. The exclusion criteria were emergency surgery, speech impairment or severe hearing or vision impairment that prevented communication, central nervous system diseases (dementia, depression), severe renal insufficiency (requiring dialysis), or participation in other relevant clinical studies within 3 months. Mini Mental State Examination (MMSE) examination confirmed the existence of cognitive dysfunction: illiteracy less than 17 points, primary school level less than 20 points, secondary school level (including technical secondary school) less than 22 points, and university level (including junior college) less than 24 points.

The primary outcome was the incidence of POD. The secondary outcomes were the 1-year incidence of adverse events, 1-year all-cause mortality, and 1-year all-cause readmission rate were evaluated.

The main outcome was the incidence of POD. The sample size was based on a literature review and early study results. Considering a 10% dropout rate, a total of 536 study volunteers, consisting of 411 patients in the non-frail group, and 125 patients in the failure group, will be included.

All the statistical analyses were performed in R v.4.0.2, and the data were analyzed using two-tailed tests.

During the study period, 810 patients aged 70 years and over who underwent non-cardiac surgery were evaluated. A total of 564 subjects were included in the study. Patients were followed up for 1 year after surgery. Among them, 441 patients completed the follow-up and 78 patients did not (Fig. 1).

**Figure 1:**
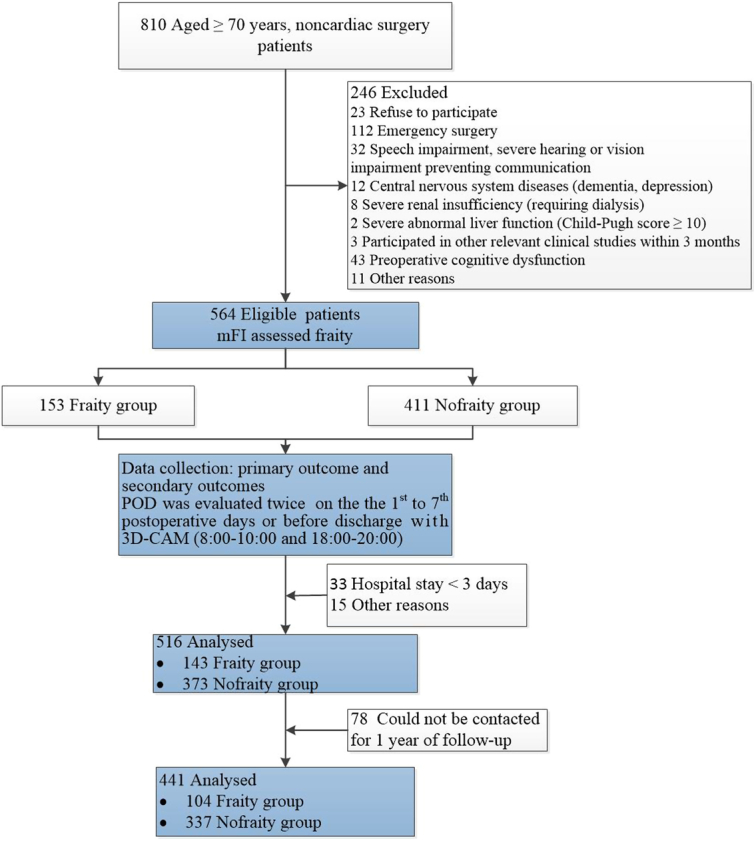
Flowchart of the study population. POD, postoperative delirium; 3D-CAM, 3-minute diagnostic confusion assessment method.

In this cohort, 143 patients (27.7%) were assessed as frail and 373 patients (72.3%) were not frail. Postoperative delirium developed in 45 (31.5%) frail patients and in 65 (17.4%) non-frail patients. The aCCI (5 (4, 7) vs. 4 (3, 5), *P* < 0.001) and the proportion of patients with an ASA physical status greater than or equal to 3 (5 (4, 7) vs. 4 (3, 5), *P* < 0.001) were significantly higher in the frail group vs. the non-frail group (Table 1). Univariate correlation analysis and multivariate regression were performed in Supplementary material 1, Supplemental Digital Content 1, http://links.lww.com/JS9/D334.

**Table 1:** Baseline characteristics.

	Frail (*n*=143)	Non-frail (*n*=373)
Age, years, median (IQR)	75 (72,78)	74 (72,77)
Sex, *n* (%)		
Male	74 (52)	189 (51)
Female	69 (48)	184 (49)
Height (cm)	165 (160, 170)	163 (158, 170)
Body weight (kg)	67 (60, 75)	66 (59, 73)
BMI, mean (SD), kg/m^2^	25.0 (22.5, 26.7)	24.5 (22.2, 27.0)
Education level, *n* (%)		
High school or above, *n* (%)	38 (26.6)	103 (27.6)
Below high school, *n* (%)	105 (73.4)	270 (72.4)
Preoperative MMSE	27 (25, 28)	27 (25, 28)
Alcohol consumption history, *n* (%)	36 (25.2)	111 (29.8)
Smoking history, *n* (%)	41 (28.7)	102 (27.4)
aCCI	5 (4, 7)	4 (3, 5)
ASA physical status ≥ 3, *n* (%)	75 (52.4)	97 (26.0)
Delirium, *n* (%)	45 (31.5)	65 (17.4)
Surgery category, *n* (%)		
Thoracic surgery	10 (7.0)	29 (7.8)
Spine surgery	26 (18.2)	16 (4.3)
Joint surgery	36 (25.2)	131 (35.1)
General surgery	41 (28.7)	120 (32.2)
Gynecological surgery	6 (4.2)	7 (1.9)
Urological surgery	15 (10.5)	50 (13.4)
Ear, nose and throat (ENT) surgery	3 (2.1)	3 (0.8)
Other	6 (4.2)	17 (4.6)
Type of anesthesia		
General anesthesia, *n* (%)	117 (81.8)	308 (82.6)
Non-general anesthesia, *n* (%)	26 (18.2)	65 (17.4)
Postoperative analgesia, *n* (%)		
Yes	104 (72.7)	237 (63.5)
No	39 (27.3)	136 (36.5)
Operative time, min (median (IQR))	123 (89, 201)	109 (85, 178)
Duration of anesthesia, min (median (IQR))	150 (112, 232)	136 (111, 219)
Intraoperative medications, *n* (%)		
Propofol	116 (81.1)	311 (83.4)
Benzodiazepines	132 (92.3)	351 (94.1)
Opioid drugs	119 (83.2)	312 (83.6)
Glucocorticoids	50 (35.0)	108 (29.0)

aCCI, age-adjusted Charlson comorbidity index; ASA, American Society of Anesthesiologists; ENT, ear, nose and throat; IQR, interquartile range; MMSE, Mini Mental State Exam.

Table 2 shows that 22 (5.0%) patients died within 1 year after surgery. An aCCI of 5 (4, 6) vs. 4 (3, 5) (*P*=0.034) and an ASA physical status greater than or equal to 3 [*n*=12 (54.5%) vs. *n*=132 (31.5%), *P*=0.025] increased all-cause mortality within 1 year after surgery. The 1-year all-cause mortality after joint surgery was the lowest [1 (4.5) vs. 142 (33.9), *P*=0.008]. The 1-year incidence of adverse events and 1-year all-cause readmission rate were performed in Supplementary material 2, Supplemental Digital Content 2, http://links.lww.com/JS9/D335.

**Table 2:** One-way correlations with postoperative 1-year all-cause mortality.

	Postoperative 1-year all-cause mortality		
	Yes (*n*=22)	No (*n*=419)	*P*
Age, years, median (IQR)	73 (71, 76)	74 (72, 77)	0.290
Sex, *n* (%)			0.117
Male	15 (68.2)	214 (51.1)	
Female	7 (31.8)	205 (48.9)	
Height (cm)	164 (158, 170)	163 (159, 170)	0.893
Body weight (kg)	67 (60, 74)	66 (60, 73)	0.766
BMI, mean (SD), kg/m^2^	24.8 (22.7, 26.6)	24.6 (22.2, 27.1)	0.778
Education, *n* (%)			0.349
High school or above	8 (36.4)	114 (27.2)	
Below high school	14 (82.5)	286 (72.8)	
Preoperative MMSE	27 (25, 29)	27 (25, 28)	0.342
Alcohol consumption history, *n* (%)	3 (13.6)	120 (28.6)	0.126
Smoking history, *n* (%)	6 (27.3)	117 (27.9)	0.947
aCCI	5 (4, 6)	4 (3, 5)	0.034
Weakness, *n* (%)	9 (40.9)	115 (27.4)	0.171
Delirium	7 (31.8)	84 (20.1)	0.184
Asthenic delirium (Fisher’s test)	3 (13.6)	34 (8.1)	0.363
ASA physical status ≥ 3, *n* (%)	12 (54.5)	132 (31.5)	0.025
Surgery category, *n* (%)			
Thoracic surgery	3 (13.6)	30 (7.2)	0.260
Spine surgery	3 (13.6)	36 (8.6)	0.417
Joint surgery	1 (4.5)	142 (33.9)	0.008
General surgery	9 (40.9)	126 (30.1)	0.282
Gynecological surgery	0	10 (2.4)	1.000
Urological surgery	4 (18.2)	52 (12.4)	0.428
ENT surgery	0 (0)	4 (1.0)	1.000
Other	2 (9.1)	19 (4.5)	0.642
Type of anesthesia			0.937
General anesthesia, *n* (%)	18 (81.8)	340 (81.1)	
Non-general anesthesia, *n* (%)	4 (18.2)	79 (18.9)	
Postoperative analgesia, *n* (%)			0.877
Yes	15 (68.2)	279 (66.6)	
No	7 (31.8)	140 (33.4)	
Operative time, min (median (IQR))	171 (85, 226)	110 (86, 176)	0.343
Duration of anesthesia, min (median (IQR))	208 (112, 275)	138 (112, 214)	0.243
Intraoperative medications, *n* (%)			
Propofol	18 (81.8)	342 (81.6)	0.982
Benzodiazepines	20 (90.9)	393 (93.8)	0.588
Opioid drugs	18 (81.8)	346 (82.6)	0.927
Glucocorticoids	8 (36.4)	123 (29.4)	0.924

aCCI, age-adjusted Charlson comorbidity index; ASA, American Society of Anesthesiologists; ENT, ear, nose and throat; IQR, interquartile range; MMSE, Mini Mental State Exam.

This cohort study showed that frailty is common among Chinese non-cardiac surgical patients aged 70 and over, with an incidence rate of ~27.6%, which is much greater than the 18.6% incidence of frailty found in the study by Elizabeth *et al.* ^6^. Frailty, as defined based on the 11-item mFI, was positively correlated with the incidence of POD and is also a recognized independent risk factor for the development of POD. According to our multivariate model, older age was associated with higher odds of POD, in line with previous studies^7^.

Although the underlying mechanism is unclear, there are many potential factors involved in the pathogenesis of POD. Our study revealed that older age, male sex, lower BMI, lower preoperative MMSE score, higher aCCI, frailty, ASA physical status greater than or equal to 3, spine surgery, postoperative analgesia, long operative duration, long duration of anesthesia, history of COPD, sensory impairment, and cerebrovascular accidents without sequelae were associated with the development of POD, which agrees with earlier results^7^.

Frailty is a strong predictor of postoperative complications, survival rate, and length of hospital stay^8^. Our study also evaluated the long-term outcomes of patients 1 year after surgery and revealed that frailty increased them within 1 year after surgery. However, the incidence of adverse events did not increase the 1-year mortality or readmission rate after surgery. These results further elucidate the role of frailty in preoperative risk stratification. Moreover, the risk of low-pressure surgery is not low for frail patients, and there is no low-risk surgery for frail patients^9^. Twenty-two (5.0%) patients died within 1 year after surgery; aCCI [5 (4, 6) vs. 4 (3, 5), *P*=0.034] and an ASA physical status greater than or equal to 3 [*n*=12 (54.5%) vs. *n*=132 (31.5%), *P*=0.025] increased all-cause mortality1-year after surgery, in line with another study in which 40.9%, 31.8%, and 13.6% of patients with frailty, delirium, and delirium plus frailty died, respectively^10^.

In conclusion, frailty is an independent predictor of POD. Age and postoperative sedation are associated with an increased incidence of POD. Frailty also increases the incidence of adverse events within 1 year after surgery but does not increase the 1-year all-cause mortality or readmission rate. Before surgery, the assessment of frail elderly patients can predict the incidence of POD.

## Ethical approval

For the clinical study protocol, it was set in compliance with Helsinki Declaration and was provided by the Ethics Committee of the First Affiliated Hospital of Shandong First Medical University (YXLL-KY-2021 (066)), Jinan, China on 23 November 2021.

## Consent

Informed consent was obtained from all the subjects involved in the study.

## Source of funding

This work was funded by the Natural Science Foundation of Shandong Province (ZR2022MH221), and Shandong Medical Association Clinical Research Fund -- Qilu Special Project (YXH2022DZX02007), and Shandong Medical Association Analgesic Sedation Anesthesia Optimization Special Project (YXH2022ZX05273).

## Author contribution

Y.S.: conceptualization, methodology, data curation, writing—review and editing, project administration, funding acquisition. T.Z.: conceptualization, supervision, investigation, resources, data curation. M.Z.: methodology, software, validation, formal analysis, writing—original draft. X.G.: software, investigation, visualization. M.L.: investigation, data curation. Z.G.: investigation, resources, data curation. Y.G.: software, investigation, project administration. L.C.: validation, formal analysis, project administration. Y.L.: conceptualization, supervision, software, investigation, project administration. X.Z.: software, investigation, project administration. L.H.: validation, investigation, project administration. M.T.: data curation, investigation, project administration.

## Conflicts of interest disclosure

The authors declare no conflicts of interest.

## Research registration unique identifying number (UIN)

1. Name of the registry: clinicaltrials.gov.
2. Unique identifying number or registration ID: NCT05189678.
3. Hyperlink to your specific registration (must be publicly accessible and will be checked): https://clinicaltrials.gov/ct2/show/NCT05189678.

## Guarantor

Yongtao Sun, Min Zhang, and Xiaojun Gao.

## Data availability statement

The datasets used and analyzed during this study are available from the corresponding author upon reasonable request. Moreover, should the editor of the journal request the data upon which the manuscript is based, I shall produce it.

## Supplementary Material
