# Supplementary material for: Correlation of preoperative frailty with postoperative delirium and 1-year mortality in Chinese geriatric patients undergoing non-cardiac surgery: a prospective observational cohort study
Source: Int J Surg. 2024 Aug 14;111(1):1576–9. doi: 10.1097/JS9.0000000000002042 (PMC11745708; doi:10.1097/JS9.0000000000002042)
Supplement: Supplementary file 1 [file js9-111-1576-s001.docx]

**Supplementary material 1**

**Tables S1** Univariate correlation analysis of postoperative delirium incidence.

|  | Delirium (n=110) | Non-delirium (n=406) | *P-*value |
| --- | --- | --- | --- |
| Age, years, median (IQR) | 75 (72, 79) | 74 (72, 77) | 0.018 |
| Sex, n (%) |  |  | 0.043 |
| Male | 66 (60.0) | 197 (48.5) |  |
| Female | 44 (40.0) | 209 (51.5) |  |
| Height (cm) | 165 (160, 170) | 163 (158, 170) | 0.155 |
| Body weight (kg) | 65 (57, 72) | 67 (60, 74) | 0.172 |
| BMI, mean (SD), kg/m^2^ | 24.2 (21.1, 26.3) | 24.7 (22.6, 27.0) | 0.036 |
| Education level, n (%) |  |  | 0.537 |
| High school or above, n (%) | 27 (24.5) | 114 (28.1) |  |
| Below high school, n (%) | 83 (75.5) | 292 (71.9) |  |
| Preoperative MMSE | 26 (24, 28) | 27 (25, 28) | 0.037 |
| Alcohol consumption history, n (%) | 36 (32.7) | 111 (27.3) | 0.322 |
| Smoking history, n (%) | 33 (30.0) | 110 (27.1) | 0.628 |
| aCCI | 5 (4, 6) | 4 (3, 5) | < 0.001 |
| Frail state, n (%) |  |  | < 0.001 |
| Weakness | 45 (40.9) | 98 (24.1) |  |
| Nondebilitated | 65 (59.1) | 308 (75.9) |  |
| ASA physical status ≥ 3, n (%) | 47 (42.7) | 125 (30.8) | 0.025 |
| Surgery category, n (%) |  |  | 0.007 |
| Thoracic surgery | 11 (10) | 28 (6.9) | 0.275 |
| Spine surgery | 15 (13.6) | 27 (6.7) | 0.018 |
| Joint surgery | 19 (17.3) | 148 (36.5) | < 0.001 |
| General surgery | 38 (34.5) | 123 (30.3) | 0.393 |
| Gynecological surgery | 2 (1.8) | 11 (2.7) | 0.852 |
| Urological surgery | 19 (17.3) | 46 (11.3) | 0.096 |
| ENT surgery | 1 (0.9) | 5 (1.2) | 1.000 |
| Other | 5 (4.5) | 18 (4.4) | 0.960 |
| Type of anesthesia, n (%) |  |  | 0.499 |
| General anesthesia | 93 (84.5) | 332 (81.8) |  |
| Partial anesthesia | 17 (15.5) | 74 (18.2) |  |
| Postoperative analgesia, n (%) |  |  | 0.005 |
| Yes | 85 (77.3) | 256 (63.1) |  |
| No | 25 (22.7) | 150 (36.9) |  |
| Operative time, min (median (IQR)) | 158 (101, 239) | 104 (83, 161) | < 0.001 |
| Duration of anesthesia, min (median (IQR)) | 198 (129, 275) | 131 (109,187) | < 0.001 |
| Intraoperative medications, n (%) |  |  |  |
| Propofol | 93 | 334 | 0.675 |
| Benzodiazepines | 100 | 383 | 0.448 |
| Opioid drugs | 95 | 336 | 0.448 |
| Glucocorticoids | 39 | 119 | 0.261 |

Abbreviations: BMI, body mass index; MMSE, Mini Mental State Exam; aCCI, age-adjusted Charlson comorbidity index; ASA, American Society of Anesthesiologists; ENT, ear, nose and throat.

**Table S2** Univariate association of mFI with POD incidence.

|  | Delirium (n=110) | Non-delirium (n=406) | *P-*value |
| --- | --- | --- | --- |
| Dependent function, n (%) | 9 (8.2) | 21 (5.2) | 0.232 |
| History of diabetes, n (%) | 28 (25.5) | 86 (21.2) | 0.338 |
| History of COPD, n (%) | 18 (16.3) | 34 (8.4) | 0.014 |
| History of heart failure, n (%) | 0 | 1 (0.2) | 1.000 |
| History of myocardial infarction, n (%) | 4 (3.6) | 6 (1.5) | 0.286 |
| After PCI or cardiac surgery, n (%) | 8 (7.3) | 33 (8.1) | 0.924 |
| Drug-treated hypertension, n (%) | 65 (59.1) | 221 (54.4) | 0.511 |
| Peripheral vascular disease, n (%) | 45 (40.9) | 130 (32.0) | 0.081 |
| Sensory impairment, n (%) | 36 (32.7) | 90 (22.2) | 0.022 |
| Cerebrovascular accidents without sequelae, n (%) | 25 (22.7) | 59 (14.6) | 0.039 |
| Cerebrovascular accidents with sequelae, n (%) | 4 (3.6) | 13 (3.2) | 0.821 |

COPD, chronic obstructive pulmonary disease; PCI, percutaneous coronary intervention.

**Table S3** Variables Associated with postoperative delirium on multivariate analysis*

|  | Postoperative delirium (n=110) | |  |
| --- | --- | --- | --- |
|  | OR 95% CI | | *P-*value |
| Age | 1.1 | 1.0-1.1 | 0.002 |
| Postoperative analgesia | 3.9 | 2.0-6.7 | < 0.001 |
| Weakness | 1.8 | 1.1-3.0 | 0.020 |

*The input variables in the regression model were age, sex, BMI, preoperative MMSE score, aCCI, frailty status, ASA status, type of surgery, postoperative analgesia, operative duration, and duration of anesthesia.
