# Supplementary material for: Correlation of preoperative frailty with postoperative delirium and 1-year mortality in Chinese geriatric patients undergoing non-cardiac surgery: a prospective observational cohort study
Source: Int J Surg. 2024 Aug 14;111(1):1576–9. doi: 10.1097/JS9.0000000000002042 (PMC11745708; doi:10.1097/JS9.0000000000002042)
Supplement: Supplementary file 2 [file js9-111-1576-s002.docx]

**Supplementary material 2**

**Tables S1** Univariate correlation analysis of adverse events 1 year after surgery.

|  | Adverse events at 1 year after surgery | |  |
| --- | --- | --- | --- |
|  | Yes (n=40) | No (n=401) | *P-*value |
| Age, years, median (IQR) | 75 (72, 79) | 74 (72, 77) | 0.018 |
| Sex, n (%) |  |  | 0.006 |
| Male | 29 (72.5) | 200 (49.9) |  |
| Female | 11 (27.5) | 201 (50.1) |  |
| Height (cm) | 165 (160, 170) | 163 (158, 170) | 0.530 |
| Body weight (kg) | 67 (58, 75) | 66 (60, 73) | 0.767 |
| BMI, mean (SD), kg/m^2^ | 25.0 (21.9, 26.8) | 25.5 (22.5, 27.0) | 0.962 |
| Education level, n (%) |  |  | 0.132 |
| High school or above | 7 (17.5) | 115 (28.7) |  |
| Below high school | 33 (82.5) | 286 (71.3) |  |
| Preoperative MMSE | 28 (26, 29) | 27 (25, 28) | 0.050 |
| Alcohol consumption history, n (%) | 14 (35.0) | 108 (26.9) | 0.193 |
| Smoking history, n (%) | 15 (37.5) | 109 (27.2) | 0.155 |
| aCCI | 5 (4, 6) | 4 (3, 5) | 0.058 |
| Weakness, n (%) | 19 (47.5) | 105 (26.2) | 0.004 |
| Delirium | 6 (15) | 85 (21.2) | 0.356 |
| Asthenia with delirium | 4 (10) | 33 (8.2) | 0.700 |
| ASA physical status ≥ 3, n (%) | 22 (55.0) | 122 (30.4) | 0.002 |
| Surgery category, n (%) |  |  |  |
| Thoracic surgery | 3 (7.5) | 31 (7.7) | 0.958 |
| Spine surgery | 2 (5.0) | 37 (9.2) | 0.369 |
| Joint surgery | 11 (27.5) | 132 (32.9) | 0.485 |
| General surgery | 12 (30.0) | 122 (30.4) | 0.956 |
| Gynecological surgery | 1 (2.5) | 9 (2.2) | 1.000 |
| Urological surgery | 9 (22.5) | 47 (11.7) | 0.051 |
| ENT surgery | 0 | 4 (1.0) | 1.000 |
| Other | 2 (5.0) | 19 (4.7) | 0.941 |
| Type of anesthesia |  |  | 0.020 |
| General anesthesia, n (%) | 27 (67.5) | 331 (82.5) |  |
| Nongeneral anesthesia, n (%) | 13 (32.5) | 70 (17.5) |  |
| Postoperative analgesia, n (%) |  |  | 0.101 |
| Yes | 22 (65.0) | 272 (67.8) |  |
| No | 18 (45.0) | 129 (32.2) |  |
| Operative time, min (median (IQR)) | 103 (84, 159) | 112 (87, 183) | 0.477 |
| Duration of anesthesia, min (median (IQR)) | 127 (110, 185) | 143 (112, 220) | 0.251 |
| Intraoperative medications, n (%) |  |  |  |
| Propofol | 27 (67.5) | 333 (83.0) | 0.016 |
| Benzodiazepines | 39 (97.5) | 374 (93.3) | 0.295 |
| Opioid drugs | 27 (67.5) | 337 (84.0) | 0.009 |
| Glucocorticoids | 12 (30.0) | 119 (29.7) | 0.966 |

Abbreviations: BMI, body mass index; MMSE, Mini Mental State Exam; aCCI, age-adjusted Charlson comorbidity index; ASA, American Society of Anesthesiologists; ENT, ear, nose and throat.

**Table S2** Univariate correlations with the readmission rate one year after surgery

|  | | Readmission within 1 year after surgery | |  |
| --- | --- | --- | --- | --- |
|  | Yes (n=103) | No (n=338) | *P-*value |  |
| Age, years, median (IQR) | | 74 (72, 77) | 74 (72, 77) | 0.979 |
| Sex, n (%) | |  |  | 0.733 |
| Male | | 55 (53.4) | 174 (51.5) |  |
| Female | | 48 (46.6) | 164 (48.5) |  |
| Height (cm) | | 164 (158, 170) | 163 (159, 170) | 0.893 |
| Body weight (kg) | | 67 (60, 74) | 67 (60, 74) | 0.766 |
| BMI, mean (SD), kg/m^2^ | | 24.8 (22.7, 26.6) | 24.6 (22.2, 27.1) | 0.778 |
| Education, n (%) | |  |  | 0.899 |
| High school or above | | 29 (28.2) | 93 (27.5) |  |
| Below high school | | 74 (71.8) | 245 (72.5) |  |
| Preoperative MMSE | | 27 (25, 29) | 27 (25, 28) | 0.107 |
| Alcohol consumption history, n (%) | | 26 (25.2) | 97 (28.7) | 0.494 |
| Smoking history, n (%) | | 31 (30.1) | 92 (27.2) | 0.569 |
| aCCI | | 5 (4, 6) | 4 (3, 5) | < 0.001 |
| Weakness, n (%) | | 34 (33.0) | 90 (26.6) | 0.207 |
| Delirium | | 18 (14.5) | 73 (21.6) | 0.365 |
| Asthenia with delirium | | 11 (10.7) | 26 (7.7) | 0.338 |
| ASA physical status ≥ 3, n (%) | | 42 (40.8) | 102 (30.2) | 0.045 |
| Surgery category, n (%) | |  |  |  |
| Thoracic surgery | | 11 (10.7) | 22 (6.5) | 0.159 |
| Spine surgery | | 5 (4.9) | 34 (10.1) | 0.103 |
| Joint surgery | | 21 (20.4) | 122 (36.1) | 0.003 |
| General surgery | | 43 (41.8) | 92 (27.2) | 0.005 |
| Gynecological surgery | | 2 (1.9) | 8 (2.4) | 0.800 |
| Urological surgery | | 15 (14.6) | 41 (12.1) | 0.516 |
| ENT surgery | | 1 (1.0) | 3 (0.9) | 1.000 |
| Other | | 5 (4.9) | 16 (4.7) | 1.000 |
| Type of anesthesia | |  |  | 0.937 |
| General anesthesia, n (%) | | 85 (82.5) | 273 (80.8) |  |
| Nongeneral anesthesia, n (%) | | 18 (17.5) | 65 (19.2) |  |
| Postoperative analgesia, n (%) | |  |  | 0.111 |
| Yes | | 62 (60.2) | 232 (67.6) |  |
| No | | 41 (39.8) | 106 (32.4) |  |
| Operative time, min (median (IQR)) | | 126 (90, 201) | 106 (85, 172) | 0.036 |
| Duration of anesthesia, min (median (IQR)) | | 156 (116, 230) | 136 (111, 197) | 0.057 |
| Intraoperative medications, n (%) | |  |  |  |
| Propofol | | 86 (83.5) | 274 (81.1) | 0.577 |
| Benzodiazepines | | 96 (93.2) | 317 (93.8) | 0.832 |
| Opioid drugs | | 88 (85.4) | 276 (81.7) | 0.376 |
| Glucocorticoids | | 31 (30.1) | 100 (29.6) | 0.921 |

Abbreviations: BMI, body mass index; MMSE, Mini Mental State Exam; aCCI, age-adjusted Charlson comorbidity index; ASA, American Society of Anesthesiologists; ENT, ear, nose and throat.
